# Supplementary material for: Development of a pharmacy-based HIV PrEP service delivery intervention for Washington, District of Columbia (DC): A study protocol
Source: PLoS One. 2024 Oct 16;19(10):e0311694. doi: 10.1371/journal.pone.0311694 (PMC11482685; doi:10.1371/journal.pone.0311694)
Supplement: Attachment — Submitted filename: Response to Reviewers.docx [file pone.0311694.s001.docx]

Dear reviewing team,

Thank you for taking the time to read through our manuscript, and to give constructive feedback to our team. Please see below our responses to your comments:

| **Comment from reviewers** | **Response from the authors** |
| --- | --- |
| 1. Please ensure that your manuscript meets PLOS ONE's style requirements, including those for file naming. The PLOS ONE style templates can be found at   https://journals.plos.org/plosone/s/file?id=wjVg/PLOSOne_formatting_sample_main_body.pdf and  https://journals.plos.org/plosone/s/file?id=ba62/PLOSOne_formatting_sample_title_authors_affiliations.pdf | Thank you, we have checked the guidelines and formatted our manuscript accordingly. |
| 2. We noticed you have some minor occurrence of overlapping text with the following previous publication(s), which needs to be addressed:  https://journals.plos.org/plosone/article?id=10.1371%2Fjournal.pone.0290631  In your revision ensure you cite all your sources (including your own works), and quote or rephrase any duplicated text outside the methods section. Further consideration is dependent on these concerns being addressed. | We apologize for the oversight on our end. We have revised the text to include all relevant citations and paraphrase any similar clauses. |
| 3. One of the noted authors is a group or consortium "BRIDGE research team". In addition to naming the author group, please list the individual authors and affiliations within this group in the acknowledgments section of your manuscript. Please also indicate clearly a lead author for this group along with a contact email address. | Thank you, we have updated the acknowledgements section to include all members of the research group that were not part of the author list. |
| 4. Please include your full ethics statement in the ‘Methods’ section of your manuscript file. In your statement, please include the full name of the IRB or ethics committee who approved or waived your study, as well as whether or not you obtained informed written or verbal consent. If consent was waived for your study, please include this information in your statement as well. | Thank you, we have revised our Methods to include our full ethics statement: “This study was approved by the George Washington University Institutional Review Board, and has been assigned the reference number NCR234875. Study participants will provide written informed consent prior to participation. ” |
| 5. Please review your reference list to ensure that it is complete and correct. If you have cited papers that have been retracted, please include the rationale for doing so in the manuscript text, or remove these references and replace them with relevant current references. Any changes to the reference list should be mentioned in the rebuttal letter that accompanies your revised manuscript. If you need to cite a retracted article, indicate the article’s retracted status in the References list and also include a citation and full reference for the retraction notice. | Thank you. We have added two more references which include a summary of states that have expanded pharmacy based PrEP, as was suggested by reviewer 3.  Reference 1: https://www.pharmacist.com/CEO-Blog/pharmacists-expand-access-to-prep-in-17-states  Reference 2: https://nastad.org/resources/pharmacist-authority-initiate-prep-pep-and-participate-collaborative-practice-agreements |
| Reviewer #1: The study intends to build evidence within Washington DC of factors that affect pharmacy-based PrEP using the Implementation Mapping (IM) framework. The goal of the intervention is to address the imbalances with regards to access to (unique) services for minorities that are more likely to suffer prejudice from unavailability or inaccessibility of the service. The authors point out that 71% of those affected (and living with HIV) are black men and women thus the intervention will be targeted at this cohort.  It is becoming increasingly true and common that people perceive pharmacists to be easily accessible and trustworthy as evidenced by the uptake of (expanded) services offered in pharmacies with vaccinations against COVID being a recent example where most people were more likely to get the vaccine from their pharmacist than any other healthcare professional. This means the choice of the authors to opt for a pharmacist led intervention is sound.  The authors have demonstrated their knowledge in the subject through the choice of their references which is very impressive and thorough. Thus, I am very impressed by this study protocol and I look forward to the results. | Thank you for this favorable comment, we hope that the proposed intervention can contribute to the knowledge base for collaboration with pharmacies for HIV prevention care. |
| Reviewer #2: The paper is written very well as well as the protocol as presented is good. The paper will guide research in the field in other regions such as those in the global south with much higher HIV prevalence, where the innovation may greatly enhance access to PrEP and/or PEP. | We appreciate the favorable comment, we also hope that our findings can inform interventions in other settings that could benefit from wide-reaching HIV prevention services. |
| Reviewer #3: This paper proposes a research protocol for the planning, design, implementation and evaluation of PrEP as delivered via pharmacies in Washington, DC. Noting that DC lacks enabling policy legislation to allow for such a service, the paper uses an implementation mapping framework to address gaps in the current knowledge of PrEP services in the area, starting with a modest needs assessment of key stakeholders, including 6 pharmacists, 6 current PrEP providers, 6 current PrEP users, 2 public health officials, 4 pharmacy officials, and 4 experts in pharmacy-delivered PrEP. The complete process will include, in addition to a needs assessment, theory-based methods and practical strategies, intervention components and materials, an implementation plan, and an evaluation plan. The next step is described in the last sentence in the abstract: ‘Information gathered through this needs assessment will be used to develop standard operating procedures for the introduction of pharmacy-based PrEP into community-based retail pharmacies.’  This is a timely topic of some urgency, given the ongoing transmission of HIV among vulnerable populations such as MSM, TGW, and women of color in DC. The authors are to be congratulated for a thoughtful approach to their needs assessment, and for assembling a credible list of investigators. | Thank you for the favorable over-arching comment, we have addressed additional specific feedback accordingly. |
| Intro Pg 3-5: The authors make a compelling case here and in the last paragraph on page 6 for the need for expanding access to PrEP in Washington DC and the known and potential benefits of pharmacy-based services. | Thank you for this feedback |
| Pg 4, para 1: The authors need to elaborate on the last sentence that the emerging plan will conform to local DC law regarding prescribing and dispensing PrEP by pharmacists. What would be allowed under current law, and what options might be available if pending enabling legislation is passed? Is there also an option to continue with an implementation research study in the absence of such legislation? | Thank you. We have added this sentence for further clarity: Currently in DC, although pharmacists do not have legal authority to provide oral PrEP without a prescription from a primary care provider (PCP)/PrEP provider, pharmacists can enter into a CPA with a PCP/PrEP provider. Under current DC laws, pharmacies are allowed to provide PrEP if they have Collaborative Practice Agreements with certified PrEP providers and physicians. After a prescription, a client can pick up their medication or get a refill from a participating pharmacy. CPAs afford pharmacists greater responsibility for patient assessments, order of related lab tests, drug administration, and initiation, adjustment or discontinuation of medication. If the current legislation is expanded, clients could initiate PrEP at any community pharmacy approved to offer PrEP without the need to establish CPAs with a PCP/PrEP provider. |
| Pg 5, para 1: Do pharmacies in DC currently offer HIV testing? Additional discussion on the implementation and training of staff around HIV testing is needed in the introduction and/or the discussion. | All pharmacies nationwide have an option for clients to purchase an HIV test. Thank you for the suggestion, please see the next response in regard to training and capacitating study stuff. |
| Page 5, para 2: Regarding the training needs, have the investigators reached out to the local HIV training authorities as collaborators, e.g. the regional AETC and the local health department? A bit more consideration of the practical elements in training to scale up this program is warranted. | Thank you for your suggestion. To assess the acceptability of the proposed intervention, we are speaking to some stakeholders who work at the local health department. We hope to build collaborations following the initial study as we develop a pilot intervention. On Page 10, we have written “At outset of the pilot phase, participating pharmacists and technicians will complete CDC's Ending the HIV Epidemic Continuing Pharmacy Education Programs (related to HIV testing and PrEP) online training courses (30). In addition to this training, the PrEP provider and pharmacist at Us Helping Us will provide more hands-on training on the operational aspects of PrEP service delivery. The hands-on training will allow participating pharmacies to work closely with Us Helping Us team members who have extensive experience providing PrEP services to the Black population in DC.” |
| Pg 6, first sentence: The authors should clarify the rationale for targeting a single vulnerable group, i.e. black adults, and the approach and applicability of the service implementation plan for other at-risk individuals. If an inclusive approach based on HIV risk is to be pursued, as well as a focus on reaching black adults in DC, that should be stated. | Thank you for your comment. While multiple groups of marginalized individuals and communities could benefit from this intervention, we are working with our community partner to tailor PrEP services to this particular sub-population, Black adults in the Washington DC area, which remains disproportionately impacted by the burden of new HIV infections |
| Pg 6, first sentence: The reference relates to telehealth in Iowa. Is there specific pending legislation in D.C. that the authors can allude to? | We apologize for the oversight. We have updated the reference. In addition, we have changed the sentence, “There is a need for a better understanding of what works and does not work, and whether it can be effective in reducing new HIV infections, particularly in the face of pending or absent legislation” to more accurately reflect that legislation to expand pharmacy-based PrEP services has been proposed and enacted in some states, but has not yet been proposed or passed in Washington DC. |
| Methods  Pg 15 – The partnership with Us Helping Us is well-conceived and described. This element in the design of the NA is critical. The specific inclusion of black women is also essential. | Thank you for your feedback. |
| Pg 8 – Intervention development: The authors should address how they will assess capacity for the PrEP work among the pharmacy interviewees and in the affected communities. Will there be an effort to generate public and/or private funding from this effort? On page 9, the authors indicate an interest in developing a research program with R01 funding to support the test of the efficacy of the service that is developed. What alternatives would be available if the R01 were not funded? | This is a helpful comment. We plan to work with Us Helping Us to apply for various funding opportunities (e.g., NIH, Gilead Sciences, Gates). We have deleted the specific mention of R01 as there are many potential opportunities. |
| The iterative generation of plans and then a second review by the planning group is important for refinement and modification of the plan. | We fully agree. |
| Results: The plan for a needs assessment and the overall plan to develop pharmacy-based PrEP are offered as results for this paper. | That is correct. |
| Discussion The authors expect, ‘at a minimum’, that the study will lead to an intervention prototype and tools, technical and educational products, and further scientific papers on the method and outcome. The authors would be well advised to focus on the substance of the paper, i.e. the needs assessment and its strengths and limitations, as well as the key next steps for deploying PrEP in pharmacies in black communities in DC, rather than offering promises of future actions that may or may not take place. | Thank you. We have added in the discussion that as a next step, we will pilot the developed intervention in a small number of pharmacies in DC. We have also deleted the list of offerings/promises. |
| For example, the authors could use their literature review of pharmacy-based PrEP to consider likely elements of the plan and its implementation in the discussion without limiting the options that will be pursued based on the needs assessment. Do the authors imagine that each pharmacy will have a local PharmD ‘leader’ to manage the start-up and engage people at risk of HIV? Will a community liaison collaborate with the whole process, or perhaps with each participating pharmacy? If community ‘accompaneurs’ are envisioned, how will they be recruited and trained? Will they be all volunteer, or will the group seek to offer reimbursement for this key service? | We have added the following regarding the training of pharmacists for the pilot phase: “At outset of the pilot phase, participating pharmacists and technicians will complete CDC's Ending the HIV Epidemic Continuing Pharmacy Education Programs (related to HIV testing and PrEP) online training courses (30). In addition to this training, the PrEP provider and pharmacist at Us Helping Us will provide more hands-on training on the operational aspects of PrEP service delivery. The hands-on training will allow participating pharmacies to work closely with Us Helping Us staff who have extensive experience providing community-based PrEP services to the Black population in DC.” |

Kind regards,

Co-author team
